# A Decade of Teaching the Course Aging & the Arts: Reflecting on Opportunities and Challenges

**DOI:** 10.1093/geroni/igab046.264

**Published:** 2021-12-17

**Authors:** Jacqueline Eaton

**Affiliations:** University of Utah, University of Utah, Utah, United States

## Abstract

In 2010, the University of Utah Gerontology Interdisciplinary Program first offered GERON 5240/6240: Aging and the Arts. This course was developed to enrich program curricula by addressing a gap in content specific to the arts and humanities. The purpose of this presentation is to focus on identifying the opportunities and challenges experienced teaching this course over the past decade. Opportunities will highlight competency mapping, internal and external partnerships, the benefits of bridging disciplines, and innovation in teaching and problem-solving. Challenges experienced include addressing various needs (online learning, undergraduate and graduate levels, multiple disciplines), tuition differentials, and varying levels of enrollment. A stand-alone course is one method of increasing humanities, arts, and cultural gerontology within curricula. It has the potential of enhancing student interest in gerontology while also demonstrating how the arts and humanities can improve work across disciplines.

